# Stem Cell-Based Clinical Trials for Diabetes Mellitus

**DOI:** 10.3389/fendo.2021.631463

**Published:** 2021-02-26

**Authors:** Eleonora de Klerk, Matthias Hebrok

**Affiliations:** Diabetes Center, University of California San Francisco, San Francisco, CA, United States

**Keywords:** stem cells, type 1 diabetes (T1D), type 2 diabetes (T2D), clinical trial (CT), transplantation, encapsulation, islets

## Abstract

Since its introduction more than twenty years ago, intraportal allogeneic cadaveric islet transplantation has been shown to be a promising therapy for patients with Type I Diabetes (T1D). Despite its positive outcome, the impact of islet transplantation has been limited due to a number of confounding issues, including the limited availability of cadaveric islets, the typically lifelong dependence of immunosuppressive drugs, and the lack of coverage of transplant costs by health insurance companies in some countries. Despite improvements in the immunosuppressive regimen, the number of required islets remains high, with two or more donors per patient often needed. Insulin independence is typically achieved upon islet transplantation, but on average just 25% of patients do not require exogenous insulin injections five years after. For these reasons, implementation of islet transplantation has been restricted almost exclusively to patients with brittle T1D who cannot avoid hypoglycemic events despite optimized insulin therapy. To improve C-peptide levels in patients with both T1 and T2 Diabetes, numerous clinical trials have explored the efficacy of mesenchymal stem cells (MSCs), both as supporting cells to protect existing β cells, and as source for newly generated β cells. Transplantation of MSCs is found to be effective for T2D patients, but its efficacy in T1D is controversial, as the ability of MSCs to differentiate into functional β cells *in vitro* is poor, and transdifferentiation *in vivo* does not seem to occur. Instead, to address limitations related to supply, human embryonic stem cell (hESC)-derived β cells are being explored as surrogates for cadaveric islets. Transplantation of allogeneic hESC-derived insulin-producing organoids has recently entered Phase I and Phase II clinical trials. Stem cell replacement therapies overcome the barrier of finite availability, but they still face immune rejection. Immune protective strategies, including coupling hESC-derived insulin-producing organoids with macroencapsulation devices and microencapsulation technologies, are being tested to balance the necessity of immune protection with the need for vascularization. Here, we compare the diverse human stem cell approaches and outcomes of recently completed and ongoing clinical trials, and discuss innovative strategies developed to overcome the most significant challenges remaining for transplanting stem cell-derived β cells.

## Why the Need for Stem-Cell Based Therapy in Diabetes?

Intraportal allogeneic cadaveric islet transplantation is considered the best available treatment for patients with Type 1 Diabetes who cannot control their blood glucose levels with exogenous insulin, despite optimal intensive medical management. It consists of the isolation of pancreatic islets from deceased donors, and their infusion into the liver through the portal vein, which results in engraftment in the hepatic parenchyma (1).

Over two decades have passed since the first seven Type 1 diabetic patients were treated with what is known as the Edmonton Protocol (2), a procedure developed by Dr. James Shapiro and his team. Before the introduction of this ground-breaking protocol, the success rate of islet transplantation (measured as percentage of patients able to remain insulin independent for more than one year) was only 8% (3). Modifications to the standard protocol led to an unprecedent 100% success rate in the first seven patients (2). These modifications included a steroid-free immunosuppressive regimen, the use of xeno-protein-free media during islet isolation, and the immediate transplantation of the purified islets from multiple donors (mean islet mass of 11,547 ± 1,604 islet equivalents per kilogram of body weight), to reach an adequate islet mass capable of restoring normoglycemia. Despite the short-term success in maintaining insulin independence during the first year after transplantation, only 11% of those patients remained insulin-independent after five years. Further improvements to the Edmonton protocol over the last twenty years have markedly increased the safety of islet transplantation, with regard to the rate of adverse events related to the infusion procedure, and to the immunosuppression regimen.

Adverse events categorized as “possibly or definitely related” to the infusion procedure include peritoneal hemorrhage, hepatic hematoma or hemorrhage, portal vein thrombosis, and abnormal liver function, while those related to immunosuppression include leukopenia, mucosal inflammation, graft vs host disease, pneumonia, increased blood creatinine, renal disorder, skin disorder, and hypertension. Data from annual reports released by the Collaborative Islet Transplant Registry (CITR, https://citregistry.org/), which includes clinical trial data from 37 islet transplantation centers (28 in North America, seven in Europe, three in Australia) collected between 1999–2016, indicate that the rate of adverse events in the first 30 days following transplantation dropped from 66 to 22%. Reports from the most recent clinical trial data being collected are not yet publicly available. Although islet transplantation has become one of the safest and least invasive transplant procedures, it currently still requires life-long immunosuppression. In addition, long-term insulin independence, which is often reached right after transplantation, declines over time. According to the latest CITR annual report (10^th^ Annual Report, released in January 2017), the percentage of patients remaining insulin-independent after one year is approximately 50%, and a drop to 25% is observed after five years. A drop in insulin-independency was also reported by the more recent phase 3 CIT trial of human islet-after-kidney transplantation (4), where only 16.7% of the patients retained insulin independence three years after transplantation. These percentages are much lower than what was reported for patients undergoing total pancreas transplantation. Based on a report summarizing data from 2005 to 2016 (from one single transplantation center), 75% of the patients who obtained pancreas transplantation following total pancreatectomy remained insulin-independent [until their time of death, or until the present day (5)].

While these numbers indicate that certain aspects of cadaveric islet transplantation need to be further optimized to achieve long lasting relief from exogenous insulin injections, it is important to note that many clinical goals are still achieved with this procedure, especially with regards to the restoration of hypoglycemia awareness and protection from severe hypoglycemic events (6–8). Hypoglycemia unawareness, a state in which a person is unaware of inappropriately low blood glucose levels, is a severe, relatively frequent and potentially life threatening complication occurring in approximately 40% of patients with type 1 diabetes mellitus (T1DM) (9) (10).

Cell survival and graft rejection are the two key unresolved challenges for increasing insulin independence rates in islet transplantation. Currently, most standard islet transplantations are performed through infusion into the portal vein. Despite encouraging results, the liver might not be the optimal place for transplanted islets as entrapment in the hepatic vasculature results in hypoxia (11), and the revascularization process can take up to 14 days to be fully established (12). In addition to hypoxia that impairs β cell function and survival, acute graft loss is caused by instant blood-mediated inflammatory reaction (IBMIR), which results in activation of the complement cascade, clot formation, and lymphocyte recruitment (13). Together, hypoxia and IBMIR lead to destruction of more than 50% of transplanted islets in the first 48 h following infusion into the portal vein (14). Only a few alternative transplantation sites have been tested in clinical trials so far: the bone marrow (15), the muscle of the forearm (16, 17), and the omentum (18–20). Despite positive outcomes for autologous islet transplantation, clinical trials have shown that the bone marrow site is not a suitable alternative site for pancreatic islet allotransplantation in T1D patients, due to recurrence of autoimmunity (21). Survival of alloislets in the intramuscular site has also been shown to be limited so far (17). The omentum represents a promising site, but the protocols utilizing this site need further optimizing to ensure better vascularization and improved management of immunosuppression for long term success (19).

Risks associated with life-long immune suppressive drugs, together with the limited availability of cadaveric islets, are the two prominent current obstacles to a broader use of islet transplantation for the treatment of diabetes. Immune suppression in general remains critical to prevent rejection of the graft. A combination of induction (administered only at the time of transplant) and maintenance (administered for long-term regime) immune suppressive agents are necessary for graft survival, and despite the advancements in the immunosuppressive regimen (22), the majority of the patients still require additional islet infusions (10^th^ Annual Report, CITR).

Islet transplantation is a government-funded, standard-of care therapy in Canada, Europe, China, and other parts of Asia (23), but only for a minority of T1D patients suffering from glycemic lability, hypoglycemia unawareness, severe hypoglycemic episodes, and/or diabetic ketoacidosis, despite optimal intensive medical management. These extreme scenarios are often referred to as “brittle” diabetes. Given the limited supply of cadaveric islets, most transplant centers also limit enrollment only to T1D patients who have complete loss of C-peptide production (24). Even when islet transplantation is government-funded, access to this procedure may still be problematic. In Canada, while islet transplantation is available in the province of Alberta, access to it is more difficult in all the other provinces, where the procedure is not always recognized as a non-research therapy (25). Access to islet transplantation is highly restricted in the United States where the procedure is not approved by health insurance companies as it is still considered an experimental treatment and requires filing of an investigational new drug application (NDA) with the Food and Drug Administration (FDA). This is one of the possible reasons why the total number of centers performing islet transplantations and the total number of transplantations performed per year have declined in the US since 2014, according to the 10^th^ Annual CITR Report. Interestingly, this decline is evident not only for centers in North America, but also in the European and Australian centers that are part of the CITR. The exact reasons for this reduced activity for these centers need to be determined, but American Centers have observed a reduction in pancreas donors since the mid-2000s (https://optn.transplant.hrsa.gov/data/view-data-reports/national-data/#). Unfortunately, among all organs isolated for transplantation the pancreas has become the organ with the lowest donation rate [approximately 11 donors per 100 eligible deaths were recorded in 2018 (26)].

A curated review of islet transplant trials registered on ClinicalTrials.gov (24) also revealed that although there are a number of newly registered trials focusing on testing alternative implant sites and innovative approaches to reduce graft rejection, including encapsulation devices and immune modulators, the overall number of clinical trials for cadaveric islet transplantation is not growing.

While it is highly likely that cell survival and graft rejection will continue to improve in the future, the low supply of cadaveric islets remains the critical limitation prohibiting wide-spread use of this therapy. In contrast, the prevalence of patients with T1D is increasing globally (27). Based on a recent diabetes forecasting model (28), by 2030 the total number of people with T1D and T2D in the United States alone will increase to 50 million, a 54% increase from 2015.

One possible pathway to a treatment, and perhaps a cure, for a broad number of diabetic patients, would be access to an alternate, unlimited source of insulin-producing cells that can reconstitute physiological glucose homeostasis, eliminating the reliance on organ donors.

Stem cell-derived β-cell therapy overcomes the barrier of limited donor availability, while also possibly representing a more cost-effective therapy compared to exogenous insulin. Although the future cost of stem cell-derived β-cell therapy is unknown, a speculative cost-effectiveness analysis from an early health technology assessment study (29) calculates that 8–9 years after transplantation both cadaveric islet transplantation and stem cell-derived β-cell transplantation would be more cost-effective than exogenous insulin therapy. This calculation assumes that the manufacturing costs of stem cell-derived β cells will be similar to the costs necessary to isolate cadaveric islets. In both cases, successful long-term engraftment is essential for the therapies to become profitable (30). The cost of stem cell-derived β-cell therapy will depend on a number of variables, including the requirement of immunosuppression, the duration of graft survival, and, most importantly, the optimization of the manufacturing process (31). While avoidance of autoimmune rejection will reduce the price, higher upfront costs are needed for the development of a scalable manufacturing process and the creation of stem cell banks for clinical use. Emerging technologies ]such as stirred suspension bioreactor culture, wave bag bioreactor culture, multiplate culture, and roller bottle culture (32)] may eventually allow for mass production of stem cell-derived β cells and islet-like organoids. If a manufacturing expenses can be reduced, early health technology assessment studies demonstrated that stem cell-derived β-cell therapy will be a cost-effective (33, 34).

In addition to endogenous cell therapies, wearable computerized devices, such as insulin pumps and closed-loop systems, present alternative options for the delivery of exogenous insulin, and clinical trials are currently testing their efficacy. Insulin pumps (and sensor-augmented insulin pumps) can be programmed to continuously deliver a basal level of insulin as well as extra doses (bolus) during mealtimes. In contrast to these pumps that require manual adjustments and input from the patient, closed-loop systems (also known as artificial pancreas or automated insulin delivery systems) constitute a combined sensing-delivery system in which an external glucose sensor directs delivery of insulin from a sensor-responsive pump guided by real-time glucose sensor readings. A recent clinical trial on T1D patients reported that closed loop systems maintain control within the near normoglycemic range up to 71 ± 12% of the time. This represents a marked improvement over sensor-augmented insulin pumps, with which the normoglycemic range is maintained up to 59 ± 14% of the time (35). Moreover, closed loop systems are also able to improve glycated hemoglobin levels (HbA1c), an indicator of long-term systemic glucose levels. Similar improvements were also noticed when comparing sensor-augmented pumps against hybrid closed-loop system (where insulin is continuously administered, except during boosts at mealtime) (36), or bihormonal closed loop systems (37) that release both insulin and glucagon. The higher efficacy obtained with the bihormonal closed loop system represents an important achievement considering that glucagon’s stability in solution is much lower than that of insulin, and that its remarkably dose-response relationship requires tight regulation of its release. A meta-analysis comparing 40 studies resulted in similar finding (38), asserting that closed loop systems are more efficient than any other insulin pump therapies, and represent an efficacious and safe approach for management of T1 diabetes. While closed loop systems clearly constitute a marked improvement in blood glucose control, there remains room for improvement of the algorithms controlling insulin release, for instance during physical activity. Variables such as the duration and intensity of physical exercise, and the proper timing of hormone release relative to food intake remain hard to control with a fully automated closed-loop system (39, 40). Other concerns regard the wearability, and the cost-effectiveness, especially if considering the constant rise in the price of insulin (a 200% increase from 2002 to 2013 (41), and a 14% annual increase from 2012 to 2018 (https://healthcostinstitute.org/)).

The technical advances described above have dramatically improved the lives of patients with diabetes. However, the limitations of these systems, including need for attaching external devices that penetrate the skin and thus raise the chance of infection and scarring over time, the dependence on fully functional pumps and sensors whose dysfunction can result in rapid and life threatening changes in glucose levels, and the complexities of algorithms tasked with anticipating ever changing aspects of patients metabolism, indicate that they present a powerful temporal solution but not a cure for diabetes.

## Stem Cell-Based Approaches: Protection or Restoration of β Cells Mass

A curated list of completed, active, recruiting, and suspended stem cell-based clinical trials for both T1D and T2D, registered at ClinicalTrials.gov within the last ten years, is presented in **Table 1**. The majority of the recently completed and active trials use adult mesenchymal stem cells (MSC) derived from different origins, hematopoietic stem cells, or a combination of both. Although initial studies might have suggested the possibility of generating insulin-producing cells from MSCs, clear evidence supporting this hypothesis is currently lacking. Thus, the purpose of these current trials is to understand the mechanisms of protection provided by MSCs and evaluate their efficacy, especially in modulating the immune response. In contrast, a number of publications have demonstrated the potential for human embryonic stem cells (hESCs) (42–46) and induced pluripotent stem cells (iPSCs) (43, 47) to form functional mature insulin-producing β cells, and trials with such cells address the issue of directly restoring β cell mass. Despite these efforts being very promising, only three trials have so far utilized hESCs to derive pancreatic progenitors for β cell replacement therapy. However, successful preclinical studies in non-human primates, led by Vertex Pharmaceutical and Sigilon Therapeutics, are paving the road towards more pluripotent stem cell-based clinical trials.

**Table 1:** Completed and active stem-cell based clinical trials for T1D and T2D.

Trial ID	Study start date	Sponsor and Collaborators	Cell type	Diabetes subtype	Status	Official title	Purpose of the study	Treatment method
NCT01068951	2010-06-01	Uppsala University Hospital	MSCs	T1D	Completed	Open Study to Evaluate the Safety and Efficacy of Autologous Mesenchymal Stem Cells in Treatment of Recently Diagnosed Patients With Type 1 Diabetes Mellitus	Test if development of autoimmune diabetes may be halted by the immune modulatory properties of mesenchymal stem cells	Intravenous injection of autologous mesenchymal stem cells
NCT02239354	2014-09-01	ViaCyte. California Institute for Regenerative Medicine (CIRM)	hESCs	T1D	Suspended	A Prospective, Multicenter, Open-Label, First-in-Human Phase 1/2 Study With Two Cohorts to Evaluate the Safety, Tolerability, and Efficacy of Various Doses of VC-01™ Combination Product in Subjects With Type 1 Diabetes Mellitus	Test if VC-01™ combination product can be implanted subcutaneously and maintained safely for two years. It will also test if VC-01 is an effective treatment	Subcutenous transplantation of combination product VC-01 (PEC-01 cells loaded into PEC-Encap)
NCT03920397	2015-03-01	Universidade Federal do Rio de Janeiro	MSCs	T1D	Active / Recruiting	Allogenic Adipose Derived Mesenchymal Stem Cells and Vitamin D Supplementation in Patients With Recent-onset Type 1 Diabetes Mellitus	Unspecified	Intravenous injection of adipose tissue-derived stem/stromal cells and oral Cholecalciferol supplementation
NCT04078308	2015-07-06	Royan Institute. Tehran University of Medical Sciences, Iranian Stem Cell Council	MSCs	T1D	Active / Recruiting	Phase I/II Clinical Trial to Examine the Safety and Efficacy of Transplantation of Mesenchymal Stem Cells in New-onset Type 1 Diabetes Patients	Modulate immune response and improve regeneration	Intravenous injection of autologous mesenchymal stem cells
NCT02940418	2017-02-19	Sophia Al-Adwan	MSCs	T1D	Active / Recruiting	The Use of Mesenchymal Stromal Cells (MSC) in Type 1 Diabetes Mellitus in Adult Humans: Phase I Clinical Trial	Unspecified	Intravenous injection of allogenic adipose-derived mesenchymal cells with autologous bone marrow mononuclear cells
NCT03162926	2017-07-05	ViaCyte	hESCs	T1D	Completed	An Open-Label Study Evaluating the Safety and Tolerability of VC-02™ Combination Product in Subjects With Type 1 Diabetes Mellitus	Test if VC-02™ combination product can be implanted subcutaneously and maintained safely for up to four months	Subcutenous transplantation of combination product VC-02 (PEC-01 cells loaded into PEC-Direct). Up to six VC-02-20 implants
NCT03163511	2017-07-06	ViaCyte. California Institute for Regenerative Medicine (CIRM), Horizon 2020 - European Commission	hESCs	T1D	Active / Recruiting	An Open-Label, First-In-Human Study Evaluating the Safety, Tolerability, and Efficacy of VC-02™ Combination Product in Subjects With Type 1 Diabetes Mellitus and Hypoglycemia Unawareness	Test if VC-02™ combination product can be implanted subcutaneously and maintained safely for up to two years. It will also test if VC-02 is an effective treatment	Subcutenous transplantation of combination product VC-02 (PEC-01 cells loaded into PEC-Direct). Cohort 1: up to six VC-02-20 implants and up to two VC-02-300 implants. Cohort 2: up to ten VC-02-300 and up to two VC-02-20.
NCT03406585	2017-11-28	NextCell Pharma Ab	MSCs	T1D	Active / Recruiting	A Double-blinded, Randomized, Placebo-controlled Trial With Wharton's Jelly Derived Allogeneic Mesenchymal Stromal Cells (WJMSCs) for Preserving Endogenous Insulin Production in Adult Patients Diagnosed for Type 1 Diabetes	Unspecified	Transplantation of cell suspension with expanded allogenic MSC's procured from donated Wharton's Jelly from umbilical cord tissue
NCT03912480	2019-01-05	CAR-T (Shanghai) Biotechnology Co., Ltd.	MSCs	T1D	Active / Recruiting	Study on the Efficacy and Safety of Stem Cells From Human Exfoliated Teeth in Treating Diabetic Patients With Significantly Reduced Islet	Unspecified	Intravenous drip of dental pulp mesenchymal stem cells
NCT03973827	2019-05-17	NextCell Pharma Ab	MSCs	T1D	Active / Recruiting	An Open Label, Parallel Single Center Trial of Wharton's Jelly Derived Allogeneic Mesenchymal Stromal Cells Repeatedly Treated to Preserve Endogenous Insulin Production in Adult Patients Diagnosed With Type 1 Diabetes	Investigate safety and tolerance after a repeated allogeneic infusion of WJMSCs intravenously after one year following the repeated treatment.	Transplantation of cell suspension with expanded allogenic MSC's procured from donated Wharton's Jelly from umbilical cord tissue
NCT04061746	2020-02-13	Medical University of South Carolina. National Institute of Diabetes and Digestive and Kidney Diseases (NIDDK)	MSCs	T1D	Active / Recruiting	Cellular Therapy for Type 1 Diabetes Using Mesenchymal Stem Cells	Determine the safety and efficacy of allogeneic umbilical cord-derived mesenchymal stromal cells for the treatment of new-onset T1D and to understand the mechanisms of protection	Intravenous injection of autologous mesenchymal stem cells
NCT01719640	2011-01-01	Fuzhou General Hospital	MSCs	T2D	Completed	Autologous Bone Marrow Mesenchymal Stem Cell and Bone Marrow Mononuclear Cell Infusion in Type 2 Diabetes Mellitus	Provide signals for regeneration and improve recovery from inflammation-induced lesion	Intra-arterial pancreatic infusion of autologous bone marrow mononuclear cells in combination with autologous bone marrow mesenchymal stem cells
NCT01576328	2012-04-01	Mesoblast, Ltd.	MSCs	T2D	Completed	A Randomized, Placebo-Controlled Dose-Escalation Study to Assess the Safety and Tolerability of a Single Intravenous Infusion of Allogeneic Mesenchymal Precursor Cells (MPCs) in Patients With Type 2 Diabetes Sub-optimally Controlled on Metformin	Assess safety and tolerability of a single intravenous infusion of three doses of Mesenchymal Precursor Cells	Single intravenous infusion of MPCs
NCT01759823	2012-12-01	Postgraduate Institute of Medical Education and Research	MSCs	T2D	Completed	Efficacy and Safety of Autologous Bone Marrow Derived Stem Cell Transplantation in Patients With Type 2 Diabetes Mellitus	Unspecified	mesenchymal stem cell will be injected into superior pancreatic duodenal artery
NCT03343782	2017-11-01	Vinmec Research Institute of Stem Cell and Gene Technology	MSCs	T2D	Completed	Outcomes of Expanded Autologous Bone Marrow-derived Mesenchymal Stem Cells Therapy in Type 2 Diabetes	Evaluate safety and effectiveness of autologous bone marrow-derived mesenchymal stem cells transplantation	Transplantation of autologous bone marrow-derived mesenchymal stem cells
NCT03943940	2019-04-24	Van Hanh General Hospital	MSCs	T2D	Active / Recruiting	A Preliminary Safety and Efficacy Evaluation of Bone Marrow Mononuclear Cells (BM-MNCs) and Umbilical Cord Tissue-derived Mesenchymal Stem Cells (UC-MSC) Infusion for Type 2 Diabetes Mellitus (T2DM) Patients	Unspecified	Intravenous injection of autologous bone marrow mononuclear cells and allogeneic umbilical cord tissue-derived mesenchymal stem cells

## Mesenchymal Stem Cell-Based Therapy

Mesenchymal stem cells (MSCs), or stromal stem cells, are currently the most widely used stem cells in clinical trials (www.clinicaltrials.gov). MSCs are multipotent adult stem cells that can be derived from both adult and neonatal tissues. Although adult bone marrow is the most prevalent source, MSCs can be obtained from almost all tissues that include a perivascular area (48, 49). MSCs are derived from the mesodermal germ layer and have a trilineage differentiation potential, that is the ability to differentiate *in vitro* into osteoblasts (bone tissue), chondroblasts (cartilage), and adipocytes (fat tissue) 29. A number of studies have also shown neuronal crest-derived MSCs (50–52) and given the high heterogeneity of MSCs it remains to be determined if additional sources besides the paraxial mesoderm and the neural crest exist. From a regulatory perspective, MSCs have been classified as an advanced therapy medicinal product (https://www.ema.europa.eu/en/human-regulatory/overview/advanced-therapy-medicinal-products-overview).

**Figure 1:**
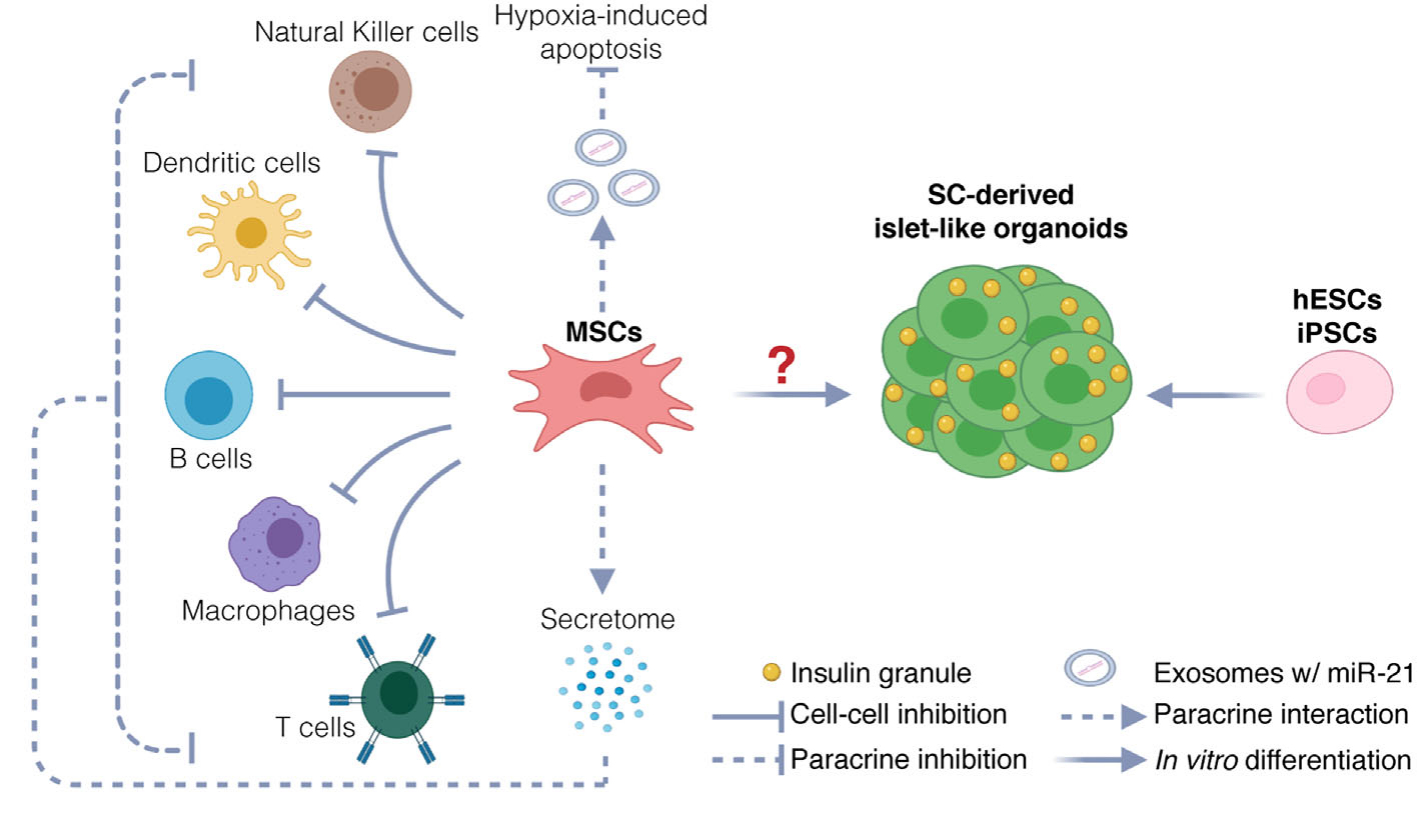
Potential therapeutic mechanisms. Potential mechanisms include protection of endogenous islets and restoration of β cells mass. MSCs could protect endogenous β cells *via* immunomodulation and inhibition of hypoxia-induced apoptosis. Immunomodulation is exerted *via* two mechanisms: inhibition through direct cell-cell interaction with immune cells, and inhibition through paracrine activity, by secretion of chemokines, cytokines, and growth factors (secretome). Inhibition of hypoxia-induced apoptosis could be exerted through release of exosomes carrying miR21, targeting messenger RNAs involved in the hypoxia-mediated ER stress preceding apoptosis. The therapeutic use of MSCs as source for generating stem-cell derived β cells and islet-like organoids is uncertain. hESCs and iPSCs instead are used to generate functional islet-like organoids to restore β cell mass.

Although MSCs are emerging as the most promising source for allogeneic cell therapy (53), the therapeutic use of MSCs in T1D clinical trials is highly controversial. Three different hypotheses have been explored in clinical settings: (a) the use of MSC-derived pancreatic progenitors that develop into functional β cells capable of restoring normoglycemia, (b) the use of undifferentiated MSCs to generate β cells through direct transdifferentiation *in vivo* upon transplantation, and (c) the use of undifferentiated MSCs to support islet health and survival without differentiating into pancreatic progenitors (**Figure 1**). As of yet, strong evidence to support the hypothesis that MSCs can differentiate into functional mature β cells or islet-like organoids, both *in vitro* and *in vivo*, is lacking.

## MSCs’ Mechanisms of Action

Early studies have investigated the hypothesis that MSCs differentiate into insulin-producing cells (54–61). This was in part based on the observation that expression of insulin and other pancreatic transcription factors increase in differentiating MSCs. However, the mere presence of such markers, including PDX1, NGN3, NEUROD1, NKX6.1, and ISL, is not proof of fully matured β cells, as some of these factors are found to be expressed also upon expansion of MSCs *in vitro* (58), and during development of other cell types, such as neurons (62). Furthermore, the presence of these proteins alone does not guarantee mature β cell activities, as expression of non-β cell factors could interfere with critical processes, whereas expression of other markers essential for mature function (including, but not limited to, K^+^-channels, Ca^2+^-channels, secretory vesicles) might still be missing. Functionality of MSC-derived insulin-producing cells has been tested by glucose stimulated insulin secretion *in vitro* and by glucose tolerance in mice *in vivo*. The claimed ability to respond to glucose by secretion of insulin may have been overestimated as insulin levels, rather than the more appropriate C-peptide levels (57, 60, 61), were measured. Insulin secretion may not indicate true insulin production as the hormone is often present in culture media (63). This is supported by the observation that when both insulin and C-peptide levels were determined, a significant increase in secretion was observed only for insulin (56) but not for C-peptide, a normal by-product generated during the maturation of the hormone. Nevertheless, one study showed a four-fold increase in C-peptide secretion upon glucose stimulation *in vitro* (58), suggesting that some MSCs may differentiate into glucose-responsive insulin secreting cells. Glucose tolerance tests have shown some ability in decreasing blood glucose levels upon transplantation into pancreactomized or Streptozotocin (STZ)-induced diabetic mice, although at suboptimal levels. Guo et al. (60) showed an initial maintenance of low blood glucose levels, followed by a drastic increase in blood sugar just 14 days after transplantation. Thus, in the absence of human C-peptide measurements it is difficult to conclude whether the initial low blood glucose levels in mice transplanted with MSC-derived insulin-producing cells were due to the presence of human C-peptide or because of persisting mouse C-peptide, especially without a proper control (STZ-treated mice without transplanted cells). The time-window was extended in the study by Dong-Qui et al. (57), but with a less favorable outcome. Although mice transplanted with MSC-derived insulin-producing cells showed an initial reduction in blood glucose levels by almost 50%, these mice developed tumors and became diabetic within 45 days. Tumor mass is most likely derived from undifferentiated MSCs as bone marrow-derived MSCs have shown to spontaneously transform into neoplastic cells during long-term culture *in vitro*. The neoplastic propensity is not observed in every study (58), possibly due to differences in the source of MSCs, the differentiation protocols employed, or the differentiation stage of the transplanted cells (64). Normoglycemia was also not determined in the study conducted by Kamalaveni et al. (58). Low levels of human C-peptide were detectable in response to glucose only 60 days after transplantation, and although still measurable 150 days post-transplantation, the range was highly variable and below therapeutic purposes (range 0.0–7.97 pmol/ml). Absence of pro-insulin transcript, suggesting the inefficiency in generating mature insulin-producing cells, was also observed in the study from Phadnis et al. (55). Furthermore, none of these studies has extensively investigated the transcriptome profile of the MSC-derived insulin-producing cells, their heterogeneity, and the percentages of poly-hormonal cells versus mono-hormonal cells. Neither were critical functional aspects of mature β cells analyzed, such as biphasic dynamic insulin secretion, proper calcium signaling, mitochondrial respiratory function, and induction of mitochondrial oxidative phosphorylation for glucose oxidation. Summarily, the evidence for differentiation of MSCs into β cells remains unconvincing. In addition, cell tracing studies in mice do not support direct transdifferentiation of multi-potent MSCs into pancreatic progenitors after transplantation *in vivo* (65–67).

Conversely, a number of pre-clinical studies performed over the last 15 years support the hypothesis that MSCs protect islet grafts (68–73) *via* at least two different mechanisms, improvement of cell survival, and immune-modulation. Back in 2012, Ezquer at al. (67) showed that intravenous administration of murine bone marrow MSCs in STZ-treated mice improves blood glucose levels, decreases glycated hemoglobin to levels similar to non-diabetic mice, and increases insulin total insulin levels. Fluorescence-tracing confirmed that MSCs do not differentiate into insulin producing cells, but engraft in lymphoid organs where they restore both the systemic and the local balance of regulatory T cells, increase anti-inflammatory markers such as IL13, and decrease proinflammatory markers such as IL1 beta, IL18, tumor necrosis factor alpha (TNF alpha), and MCP1. In addition, STZ-treated mice transplanted with MSCs also showed an increase in EGF, a trophic factor involved in cell survival. A recent study (74) has shown that highly proliferative bone marrow MSCs can promote autochthonous β cell regeneration *in vivo* in mice with partial pancreatomy. Higher levels of proliferation were reported based on an increase in number of bromodeoxyuridine positive cells, and a plausible mechanism points to the downregulation of the FoxO1 pathway. In addition to enhanced proliferation, MSC-treated mice also displayed an increase in EGF and total insulin content together with a decrease in interferon gamma and TNF alpha. These data suggest that at least some of the beneficial effects of MSC treatment are mediated *via* a reduction of inflammation.

## MSCs-Based Clinical Trials

Despite the lack of convincing evidence from pre-clinical studies, clinical trials have been performed testing the hypothesis that MSC-derived pancreatic progenitors generated *in vitro* maturate into β cells *in vivo*, either alone (75) or upon co-transplantation with bone marrow-derived stem cells (76). Although both trials reported positive outcomes in terms of improvement in HbA1c, in addition to an increase in serum C-peptide, a decrease in glutamic acid decarboxylase antibodies (GAD), and a decrease in exogenous insulin requirement, clear evidence for the presence of mature functioning MSC-derived β cells is still lacking. What is more likely is that the benefits of MSC transplantation derive from the immune-modulation and/or the protective role of these cells towards endogenous islets. In fact, co-infusion of MSCs-derived islet pancreatic progenitors with bone marrow-derived hematopoietic stem cells resulted in better long-term control of hyperglycemia as compared with MSC-pancreatic progenitors only (75, 76).

For the reasons listed above, the most recent trials (**Table 1**) focus on the third hypothesis: MSCs support islet health and survival *via* indirect means. Potential mechanisms of action include a paracrine effect through secretion of growth factors (77), modulation of extracellular matrix, ability to scavenge reactive oxygen species (ROS), ability to protect against hypoxia-induced apoptosis through micro RNAs (miRNAs) derived from exosomes (78), and the ability to modulate the immune system (79) (80, 81) through inhibition of T-cell proliferation and promotion of regulatory T-cells, or through interactions with other immune cell types, such as macrophages, B-cells, dendritic cells, and natural killer cells (53).

Meta-analyses of data from completed clinical trials suggest that MSCs can protect islets in T2D but the effect in T1D patients remains questionable. A clear interpretation of early MSC-based clinical trials for T1D has been challenged by the limited number of enrolled patients, and/or by the trial’s design that does not allow a proper statistical analysis. A trial conducted by Mesples and his team in 2013 (80) to test the efficacy of autologous bone marrow stem cell transplant reported improvements especially in the reduction of anti-pancreatic islet antibodies. The follow up study at 12 months showed negative value in islet cell antibodies (ICA), GAD, and insulin antibody levels, followed by an increased level of C-peptide and decreased levels of blood glucose and HbA1c. However, a decrease in blood glucose and HbA1c was also seen in the only available control patient, and the levels of C-peptide were not fully maintained in one of the two enrolled patients after 12 months. The small number of treated patients and controls rendered the interpretation of the data inconclusive. In 2015, Carlsson and his team conducted a similar trial (81) on twenty adult patients with newly diagnosed T1D. Treated patients showed preservation or even increase in C-peptide levels in response to a mixed-meal tolerance test 12 months after transplantation.

Although each of these trials suggested that MSC-based therapy promotes β cell health and function in T1D patients, systematic reviews and meta-analysis studies of controlled clinical trials are still debating their positive outcomes. A meta-analysis performed in 2018 (82), comprising 9 randomized-controlled trials and 14 self-controlled trials, concluded that the pooled effect of hematopoietic stem cells therapy, MSC-based therapy, and co-infusion of hematopoietic and multipotent MSCs, resulted in an increased C-peptide level, compared with conventional insulin therapy, whereas trials based on umbilical cord blood-derived MSCs did not reach a significance. A separate meta-analysis of 6 controlled T1D trials published in 2019 (83) showed that there was no difference in the levels of stimulated C-peptide and fasting C-peptide. The reduction in HbA1c was the only difference observed between treated and control patients. Results from ongoing randomized-controlled trials, with larger number of enrolled patients and controls, are needed to elucidate the efficacy of MSC therapy for T1D.

Contrary to the questionable benefits of MSC-based therapies for T1D, MSC-based clinical trials for T2D have shown a constant and robust efficacy. T2D MSC-based trials make use of multipotent MSCs derived from different sources (**Table 1**). From the first trial in 2009 (84) to the most recent trials (85–87) improvements have been observed in C-pep levels, HbA1c values, and reductions in the required insulin dosage. A systematic review of 10 T2D MSC-based trials confirmed a significant increase in the levels of stimulated C-peptide and fasting C-peptide (83). However, despite the large number of *in vitro* studies and *in vivo* pre-clinical studies already conducted, the exact mechanism by which MSCs improve outcomes still remains to be elucidated. Whether the discrepancy between T1D and T2D trials outcomes is caused by technical limitations relative to how the trials for T1D patients have been designed, or by differences in the etiology, needs to be also elucidated.

## Pluripotent Stem Cell-Based Clinical Trials

Human embryonic stem cells (hESCs) are pluripotent cells isolated from the inner cell mass (ICM) of the blastocyst (88). They possess self-renewal capacity, genomic stability, and can give rise to all three lineages (endoderm, mesoderm, and ectoderm). Induced pluripotent stem cells (iPSCs) (89) are generated from somatic cells by ectopic overexpression of specific transcription factors. iPSCs also have the capacity of self-renewal and differentiation potential, though their genomic stability is still questionable. hESCs and iPSCs maintain their pluripotency after expansion (90), thus fulfilling that need of unlimited supply required for therapeutic purposes. Although iPSCs are emerging as a potential alternative to hESCs, their ability to differentiate into mature pancreatic endocrine cells has not yet reached the same quality observed with hESC protocols (91).

In 2015, the first T1D patient was treated with a hESC-based pancreatic progenitor transplant in Edmonton. The study was driven by the regenerative medicine company ViaCyte, under the supervision of J. Shapiro’s team. The purpose of the trial (NCT02239354, submitted in 2014) was to test a combination of hESC-derived pancreatic progenitor cells (PEC-01) (92, 93) expected to mature into functional insulin-producing cells upon transplantation based on prior studies with surrogate animals, within an encapsulation device called PEC-Encap (VC-01) (94). The first transplantation consisted of 40 million pancreatic progenitor cells, divided into two encapsulation devices implanted subcutaneously in the abdomen, along with six smaller encapsulation devises implanted subcutaneously in the arm, serving as sentinels to be removed at different time points to follow cell survival and maturation. This first encapsulation device was designed to protect the pancreatic progenitor cells from the immune system, preventing both allogeneic (foreign organ) reaction and autoimmune rejection, eliminating the necessity of immunosuppressive drugs. The device had a semipermeable membrane that allowed exchange of molecules but not cells. VC-01 (consisting of the combination of PEC-01 cells and PEC-Encap device) was meant to be evaluated in an open-label, dose-escalating Phase 1/2 study in T1D patients with minimal insulin-producing β-cell function. The trial was suspended due to inconsistencies in cell survival and poor cell engraftment, primarily caused by a foreign body response, similar to a wound healing which clogged the membrane and prevented vascularization. This first trial indicated the necessity for optimization of the encapsulation device.

In 2017 ViaCyte launched a second 12-months trial (NCT03162926) which introduced an alternative encapsulation device (PEC-Direct, VC-02), with a modified membrane that does not provide immune protection, but allows vascularization, and therefore requires the re-introduction of immunosuppressants. No changes were performed in the type of hESC-derived pancreatic progenitors used. Successful outcomes led to the currently ongoing 2-year trial (NCT03163511), aimed at testing safety and tolerability of VC-02 implanted subcutaneously in T1D subjects with hypoglycemia unawareness. The purpose of this trial is also to test whether VC-02 is an effective treatment. PEC-01 cells were able to engraft, survive, and produce measurable C-peptide levels (95). Preliminary results from a small subset of patients (six out of 18) showed substantial engraftment of sentinel devices containing insulin positive cells (9 months after transplantation), and production of C-peptide in all patients up to 12-months (with some patients already reaching 15, 18, or 21 months). Moreover, the immunosuppression regimen prevented allogeneic and autoimmune destruction of the cells, without causing a foreign body response. The intended islet mass transplanted was intentionally insufficient to normalize HbA1c levels, therefore no data regarding the efficacy is available. Although these positive outcomes are restricted to 30% of the transplanted patients, further optimization of microencapsulation device materials might improve future outcomes. In support of this notion, a press release from Viacyte from August 2020 (https://viacyte.com/news-events/) announced a clinical phase agreement with Gore, a materials science company, for the development of a modified version of the original PEC-Encap, which has the potential to eliminate the need for immunosuppression while still allowing vascularization.

In addition to Viacyte, two other companies, Vertex Pharmaceutical and Sigilon Therapeutics, are moving forward towards clinical trials with stem cell derived beta cells. These companies are taking a different approach in terms of cell type and immune protection. Viacyte’s cells (PEC-01) (92, 93) consist of a mixture of hESC-derived multipotent pancreatic progenitors (which can differentiate into endocrine, exocrine, or ductal cells) and immature hormone-producing cells. This choice derives from the observation that immature progenitors can better overcome the inflammation initiated by the transplantation procedure (96). Vertex Pharmaceutical and Sigilon Therapeutics produce stem cell-derived islet-like organoids which lack the progenitor population. Islet-like organoids generated by Vertex have been tested in a pre-clinical study performed on non-human primates, mimicking cadaveric islet transplantation. Organoids were delivered through the portal vein, in combination with immunosuppressants. The transplanted organoids successfully engrafted in the liver and were functional over a period of 6 weeks. Although the transplanted amount did not lead to insulin independence, the study showed a 60% reduction in the required insulin dosage (International Society for Stem Cell Research (ISSCR) Annual Meeting 2019). Vertex is also developing its own macroencapsulation device, consisting of a porous membrane which allows immune protection. The first pre-clinical study in pigs showed that the device was able to confer immune protection, while still balancing cell survival and foreign body reaction (ISSCR Annual Meeting 2019). Alice Tomei and her team recently reported an alternative encapsulation strategy, termed conformal coating, consisting in a uniformly thin hydrogel layer that conforms to the islet shape. Preliminary data in mice revealed that the conformal-coated stem cell-derived islets could reverse diabetes and maintain euglycemia for more than 80 days (97). Positive results were also obtained from pre-clinical studies performed on macaques by Sigilon (98), using a different encapsulation strategy. Rather than generating islet-like organoids that aggregate solely by cell-cell interaction, the company utilize a microencapsulation technology consisting of gel-based spheres that can hold up to 30,000 cells (99). Chemical modifications on the surface of the spheres allows for immune protection. Endocrine cells within these clusters were shown to remain functional after transplantation for up to four months (98).

The timing for clinical trials has not been released, but both companies aim to bring their technology into clinical use in the near future.

It is worth noting that numerous companies are developing and clinically testing encapsulation devices that, although initially aimed at preserving human pancreatic islets, could be quickly applied to stem cell-derived therapies. An example of such a company is BetaO2 Technologies, which developed a bioartificial pancreas, called Beta-Air, designed to contain macro-encapsulated human islets together with an oxygen tank. Human islets encapsulated within an alginate-based hydrogel are protected from the immune system by a permselective membrane, and are continuously supplied with oxygen. The bioartificial pancreas was tested in patients with T1D in 2014 (NCT02064309), and the company is currently developing a second-generation device specifically adapted for stem cell-derived pancreatic clusters.

## Future Directions

After decades of MSC-based clinical trials for both T1 and T2 diabetic patients, hESCs and iPSCs-based β cell replacement therapies are finally becoming a tangible reality with the first hESC-derived islet-like organoids transplanted in T1D patients in 2014. A tremendous amount of clinical testing is now necessary to investigate the many aspects involved in stem cell transplantation, including the long-term safety of each encapsulation device, the optimal implant size to reach a therapeutic effect, and the long-term viability of the transplanted cells. Improvements of cell survival in subcutaneous and/or intramuscular space would alleviate safety concerns and allow for easier transplant monitoring. A preclinical study on non-human primates has shown that long-term survival (over 800 days) of human islets transplanted subcutaneously can be achieved when islets are mixed with a matrix, termed islet viability matrix, consisting of human collagen 1, l-glutamine, fetal bovine serum, sodium bicarbonate and medium199 (100). Similarly, numerous groups are developing drug-eluting scaffolds to modulate the immune reaction. These biomaterials have been shown to not only reduce local inflammation following transplantation (101) but also maintain long-term graft survival (102). Islet viability matrixes and drug eluting matrices/scaffolds should be tested in clinical trials, in combination with encapsulation devices. Co-transplantation of autologous non-endocrine tissues which may help cell survival and engraftment may also be considered, such as co-transplantation with parathyroid gland tissue, a method currently tested with cadaveric islets in the intramuscular space (NCT03977662).

Efforts to compare the quality of the cell mixtures required to achieve optimal metabolic control are crucial. Currently, three different cell mixtures can be generated: pancreatic progenitors, islet-like organoids, and enriched β cell clusters; of those, only the first two are explored in clinical trials. Numerous studies have shown that cell-cell communication between different endocrine cells are critical for correct glucose responsiveness and electrical coupling of stimulus with insulin secretion (103). Current ongoing clinical trials are utilizing a mixture of pancreatic progenitor cells which can differentiate and mature into endocrine, exocrine, or ductal cells *in vivo*. Although the architecture of these organoids more likely resembles that of endogenous human islets, it is still not known whether immature polyhormonal cells remain in small quantities after transplantation, and whether they may disrupt proper function over time. The use of mature enriched β cell clusters has not been tested in clinical settings, but considering that enriched β cell clusters lack other islet cell types, such aggregates may not provide optimal metabolic control either. Furthermore, human islets contain specialized β cells, termed hub cells, which have reduced β cell identity but regulate efficient islet response to changes in glucose levels (104). Enrichment strategies that aim at targeting highly insulin expressing cells may therefore exclude β cell hubs. Previous studies have shown that enriched β cell clusters that have already reached maturity *in vitro* can still continue to mature *in vivo*, and generate mono-hormonal glucagon and somatostatin positive cells (105). Whether these *in vivo* matured β cell clusters contain β cell hubs remains to be determined. Transplantation of islet-like organoids, consisting of mature β, alpha, and delta cells, which have been individually differentiated, and subsequently clustered based on endogenous percentages, has also not yet been tested in pre-clinical nor clinical settings, although protocols for the *in vitro* generation of both β and alpha cells have been optimized (32, 106). The generation of somatostatin-producing cells is currently achieved in small percentages as a bio-product of the β cell differentiation protocols (105).

Another aspect to consider is the use of iPSCs over hESCs. iPSCs have some advantages in terms of safety, at least in the sphere of alloimmunity, but from a technical perspective the time-consuming generation of iPSC lines from each single patient may pose an insurmountable economic burden. This is one of the reasons why numerous studies are currently testing alternative methods to eliminate overall immune rejection. These methods can be classified into two categories: induction of immune tolerance, and gene editing to generate ‘cloaked’ cells invisible to the immune system. Immune tolerance can be induced with tolerogenic cytokines and immunomodulatory proteins such as CTLA-4, and PD-L1 (107), whereas the generation of ‘cloaked’ cells is attempted by removal of HLA proteins, mainly through genome editing (108–110). ViaCyte, in partnership with CRISPR Therapeutics, is currently developing immune-evasive stem cell lines that combine both strategies. Approaches aimed at inducing immune protection have the potential concern of creating cells that cannot be recognized and thus eliminated by the immune system if they should become infected by a virus or should form a teratoma, a major concern in stem cell therapy. A possible solution may be provided by the introduction of inducible suicide genes, such as the inducible Caspase-9 (iC9) (111), combined with its in-frame insertion into a locus transcriptionally active in undifferentiated stem cells, such as SOX2 (112). Overall, while there are challenges that still need to be addressed, generating immune “cloaked” cells would remove the need for immune-suppressive regimen, thus broadening the applicability of stem cell therapies to treat patients afflicted by both type 1 and type 2 diabetes.

## Author Contributions

EdK wrote the manuscript with support from MH. All authors contributed to the article and approved the submitted version.

## Funding

Work in MH’s laboratory on stem cells is supported by grants from the NIH (DK105831) and the JDRF (COE-2019-860-S-B).

## Conflict of Interest

MH owns stocks/stock options in Viacyte, Encellin, Thymmune, and Minutia. MH also serves as SAB member to 1351 Thymmune and Encellin, and is co-Founder, SAB and Board member for 1352 EndoCrine and Minutia.

The remaining author declares that the research was conducted in the absence of any commercial or financial relationships that could be construed as a potential conflict of interest.
